# Supplementary material for: Experience of Service Questionnaire (ESQ) in children and adolescents: factor structure, reliability, validity, item parameters and interpretability of the parent version for practical use in Greece
Source: J Patient Rep Outcomes. 2024 Nov 8;8:128. doi: 10.1186/s41687-024-00798-6 (PMC11549257; doi:10.1186/s41687-024-00798-6)
Supplement: Supplementary file 1 — Supplementary Material 1 [file 41687_2024_798_MOESM1_ESM.docx]

# **Supplementary Material**

| 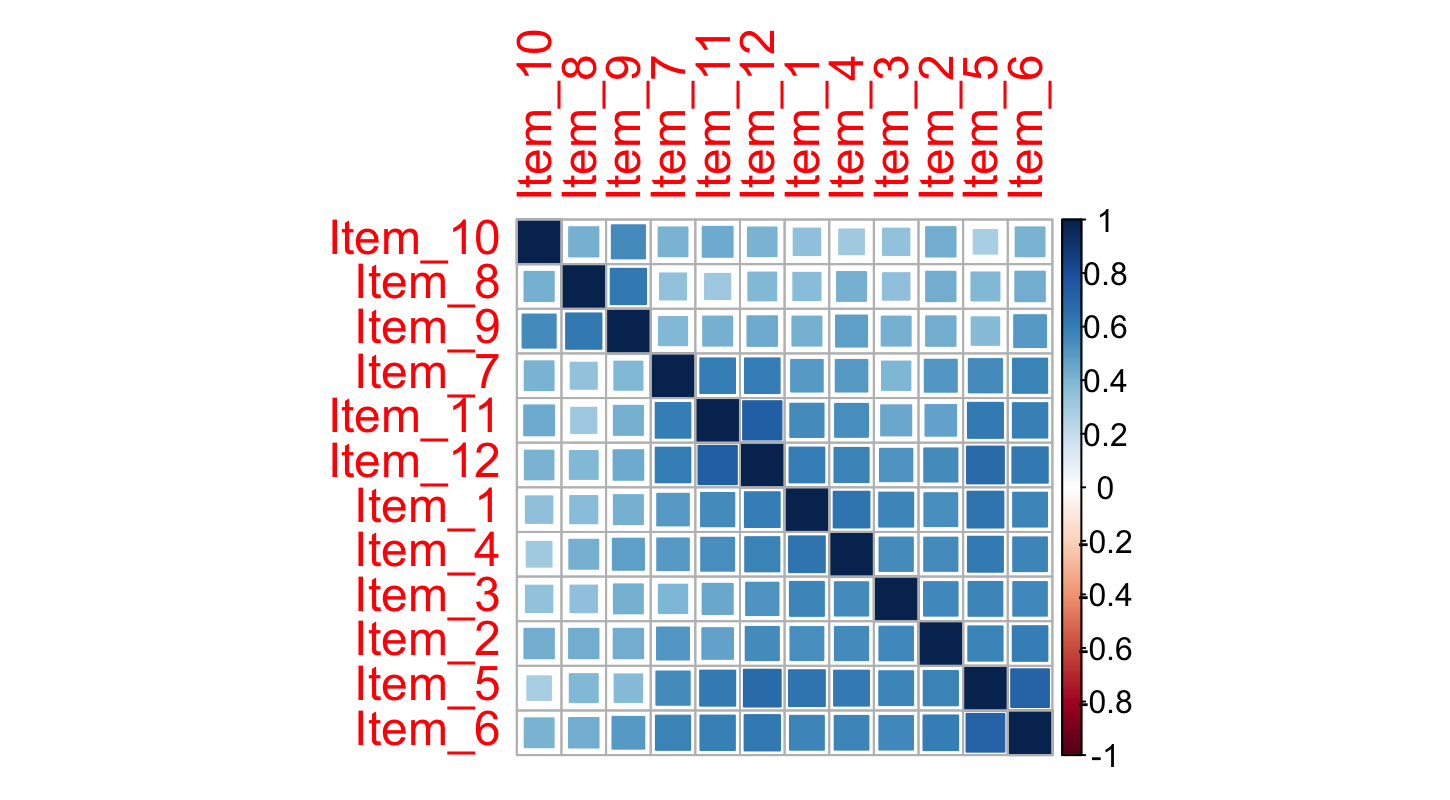 | 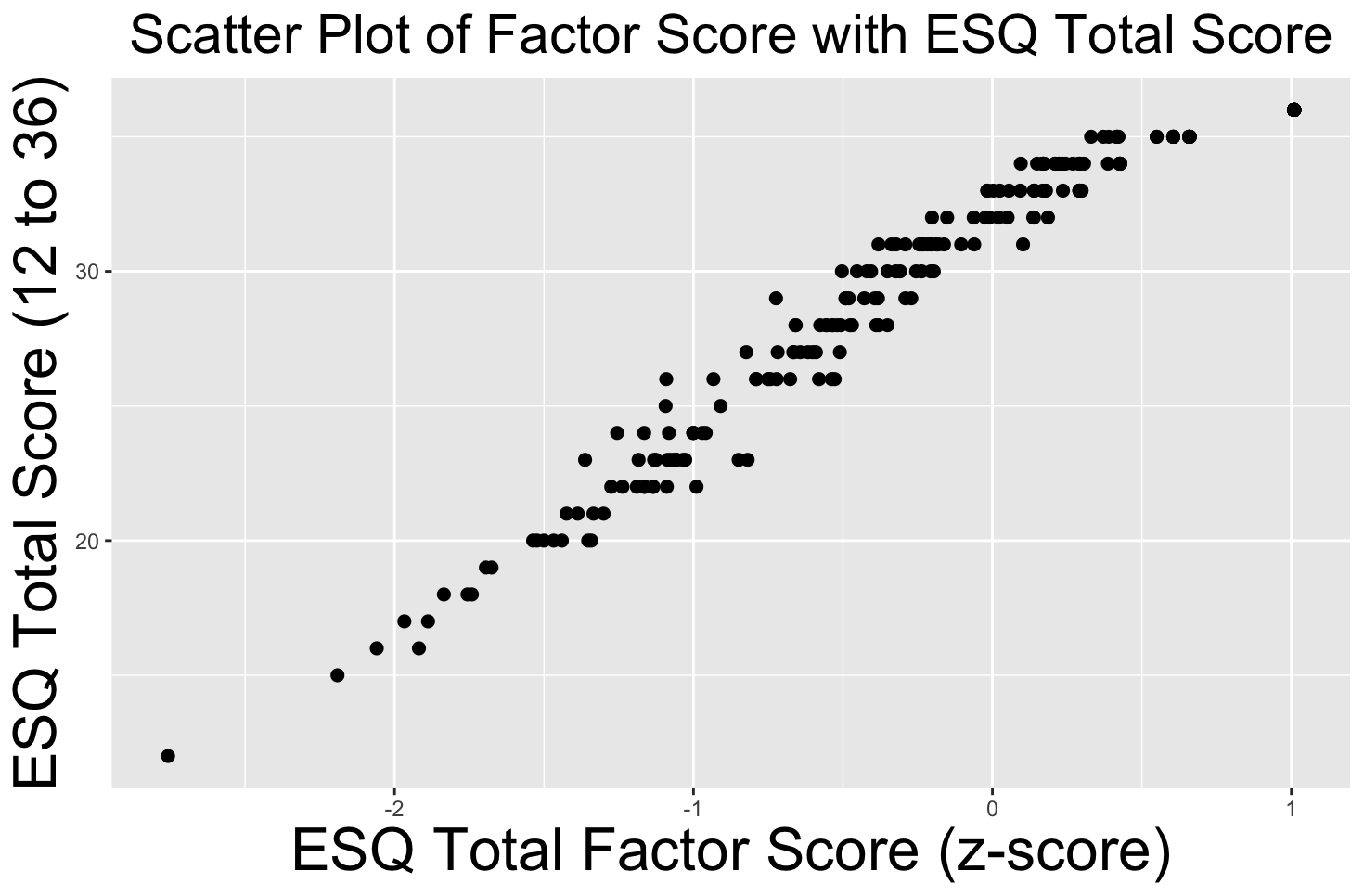 |
| --- | --- |
| 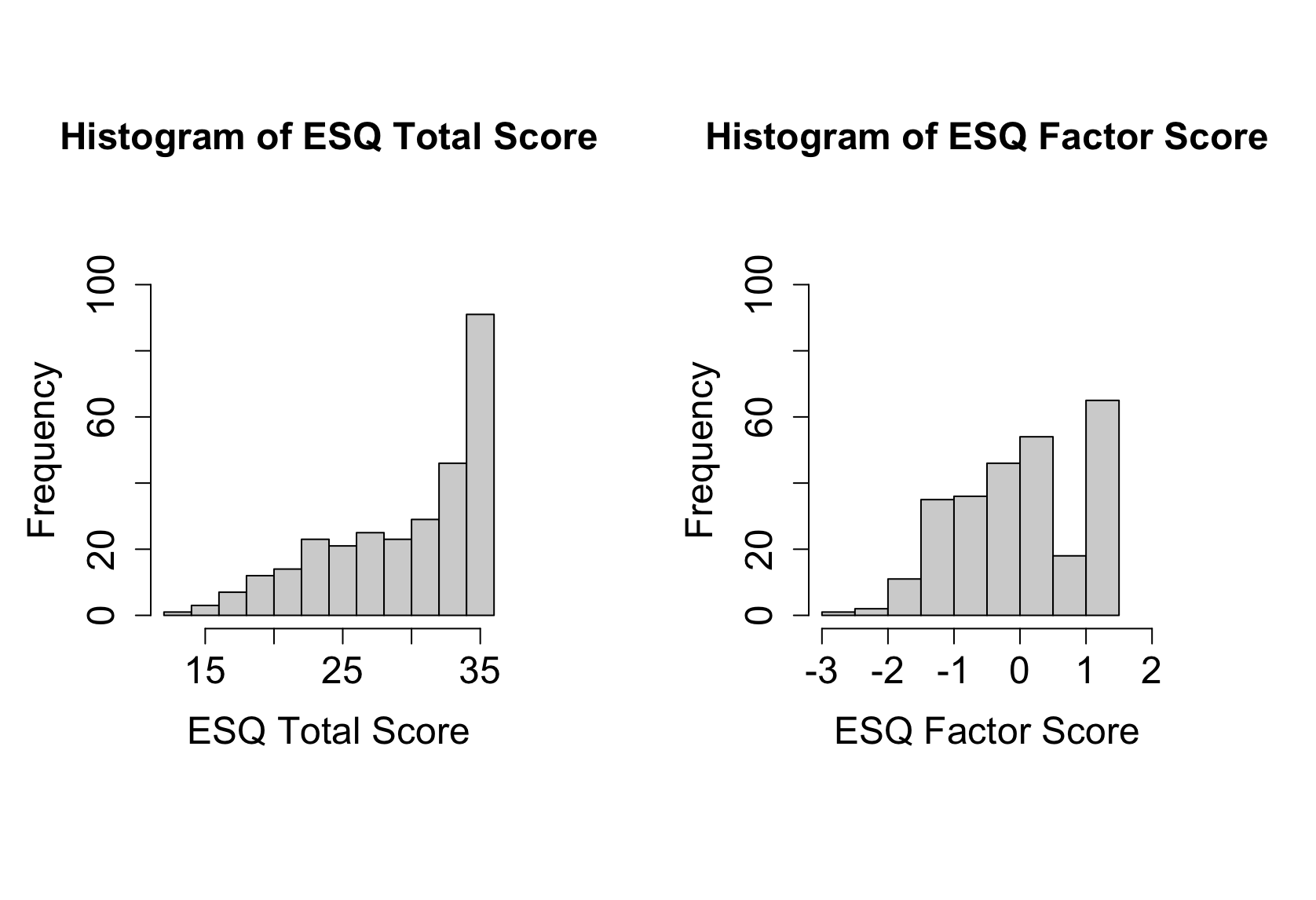 | |

#

# **Supplemental Figure S1.** Correlation matrix, histograms of the summed-based score and IRT-based score and scatter plot showing the association between summed score and IRT-based score

#

#
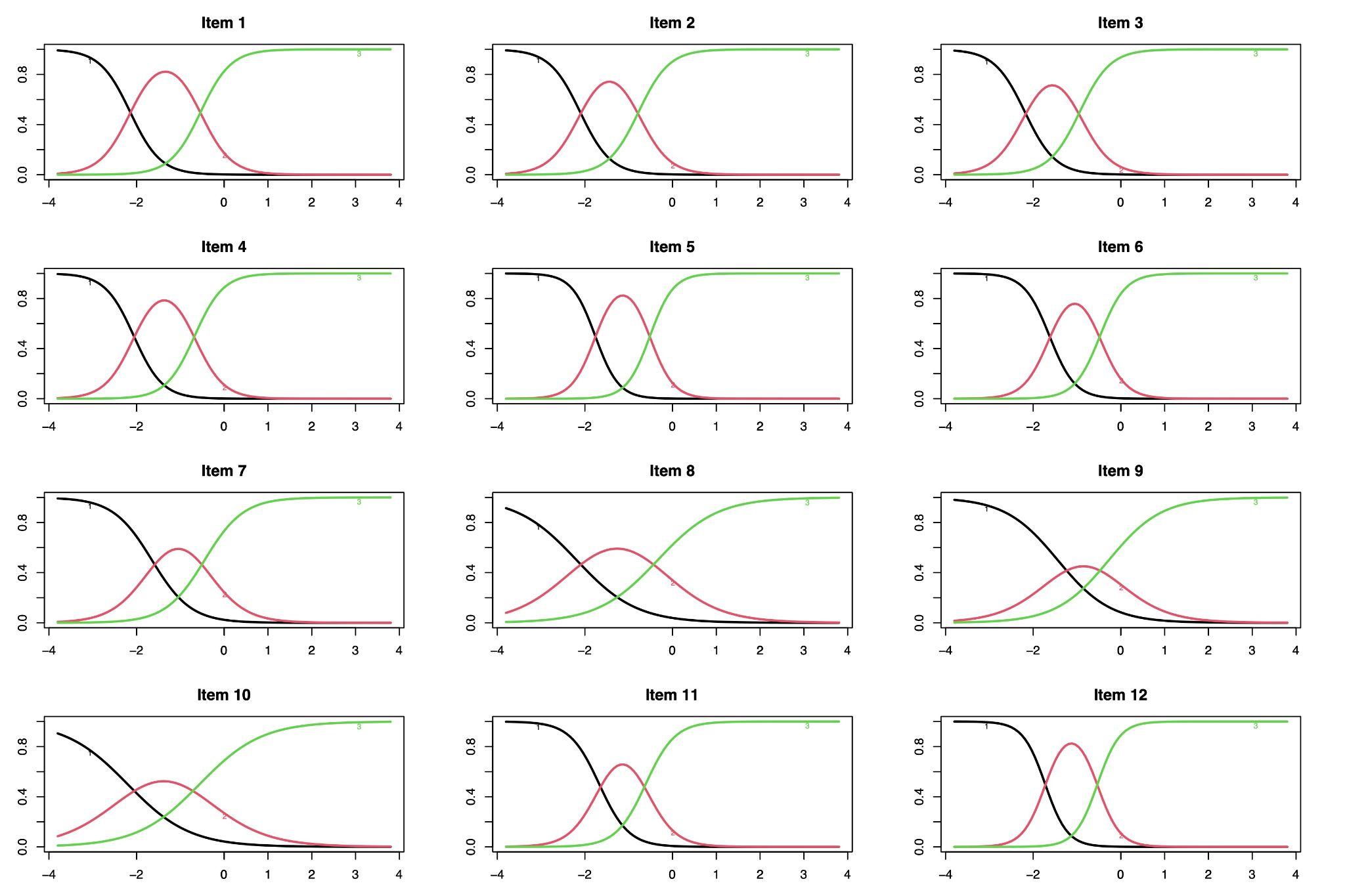


# **Supplemental Figure S2:** Item Response Characteristic Curves (unidimensional solution)


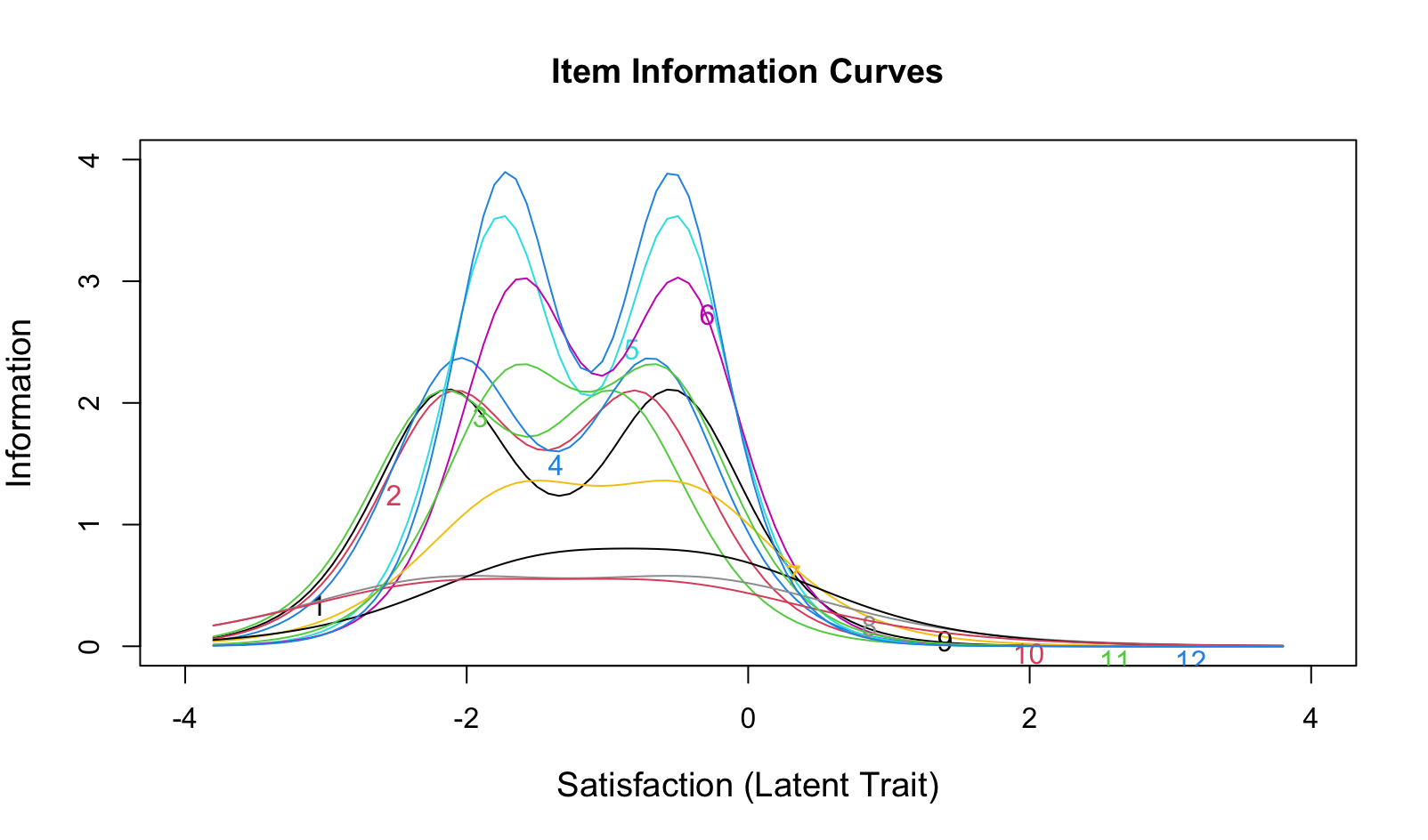


# **Supplemental Figure S3**. Item Information Curves (unidimensional solution)
